# Pivotal Factors in Breast Cancer Molecular Subtypes Apoptosis Induction by ELF‐EMF; Ki‐67, ROS Level, HER‐2, and SODs

**DOI:** 10.1155/tbj/9572421

**Published:** 2026-07-17

**Authors:** Mohadeseh Shayeghan, Fatemeh Shahriari, Fatemeh Afroughi, Flora Forouzesh, Mohammad Hadi Niakan, Mohammad Amin Javidi

**Affiliations:** ^1^ Integrative Oncology Department, Breast Cancer Research Center, ACECR, National Cancer Institute, Tehran, Iran, acecr.ac.ir; ^2^ Department of Genetics, TeMS.C., Islamic Azad University, Tehran, Iran, azad.ac.ir; ^3^ Department of Molecular Genetics, Faculty of Biological Sciences, Tarbiat Modares University, Tehran, Iran, modares.ac.ir; ^4^ Iranian Research Center for HIV/AIDS, Iranian Institute for Reduction of High Risk Behaviors, Tehran University of Medical Sciences, Tehran, Iran, tums.ac.ir; ^5^ Trauma Research Center, Shahid Rajaee (Emtiaz) Trauma Hospital, Shiraz University of Medical Sciences, Shiraz, Fars Province, Iran, sums.ac.ir

**Keywords:** apoptosis, breast cancer, ELF-EMFs, ROS level, SODs

## Abstract

**Background:**

Although increasing research has shown that extremely low‐frequency electromagnetic fields (ELF‐EMFs) specifically trigger PCD through the elevation of ROS levels in cancer cells, there is no adequate evidence to determine the exact mechanisms of this phenomenon. The antioxidant machinery may play a crucial role in this area; however, this has been neglected in previous research.

**Methods:**

The main aim of this study was to assess the effect of ELF‐EMF exposure (5 days, 1 Hz, 100 mT, 2 h/day) on ROS levels, expression levels of antioxidant genes, and apoptosis induction in different breast cancer molecular subtypes with different p53 statuses.

**Results:**

DCFH‐DA results revealed that the ROS level increased in all three cell lines (SKBR‐3, MDA‐MB‐231, and MCF‐7); this increase was much greater in SKBR‐3 (up to 5‐fold compared to its sham exposure). This result was concurrent with the annexin V/PI results; SKBR‐3 cells showed much more apoptosis induction (about 78%), compared with the others (22% or 11% in the other two cells). On the other hand, the mRNA expression level of SOD1 and SOD2 increased significantly in the MDA‐MB‐231, in addition to these two genes, the expression level of SOD3 and GSR increased in the MCF‐7 cells but not in the SKBR‐3.

**Conclusion:**

Taken together, our results confirmed that ELF‐EMF induced ROS‐dependent apoptosis, especially in HER‐2‐enriched breast cancer cells (the SKBR‐3), in a p53‐independent manner. Other molecular subtypes (MDA‐MB‐231 as TNBC, or MCF‐7 as luminal A) showed resistance against the ROS level increasing and subsequent apoptosis induction by using antioxidant genes, especially SOD1.

## 1. Introduction

Cancer is a renowned socio‐economic dilemma which has attracted many researchers worldwide. Among all known cancers, breast cancer is the most prevalent cancer in women. To increase the treatment efficiency, different techniques for early diagnosis, as well as various treatment approaches including hormone and/or targeted therapy based on cell membrane receptors in different breast cancer molecular subtypes, have developed [1]. Against all of these attempts, a sufficient outcome has not been reached, and introducing new approaches to improve the cancer treatment outcome is an ongoing task. With this point of view, extremely low frequency electromagnetic fields (ELF‐EMFs) are a noninvasive approach that, according to the literature, has the ability to inhibit cancer cells’ proliferation and various programmed cell death (PCD) induction (including apoptosis, necroptosis, and ferroptosis) in them [1–4].

A number of studies have been performed to clarify the underlying anticancer cellular and molecular mechanism of action of the mentioned fields. This is an undoubted demand prior to recruiting in these fields at the bedside. One of the most popular proposed mechanisms is due to elevation of reactive oxygen species (ROS) levels [5, 6]. Considering the therapeutic window for ROS levels in cancerous and normal cells, it is believed that as cancer cells has upper level of ROS compared with the normal ones, the former is much more susceptible to the ROS‐elevating interventions [7, 8].

Cancer cells benefit from this ROS level in various ways including mutation occurrence in them which is in some levels pivotal for these cells’ survival [9–11]. On the other hand, if this increasing at ROS level occurs unbridledly, it will induce cellular death in cancer cells. To overcome this dilemma, cells have developed ROS scavenger system which comprises different antioxidant machinery [12, 13]. Superoxide dismutases (SODs) serve as the primary line of defense enzymes against oxidative stress. They function by transforming superoxide anion into H_2_O_2_ and oxygen within the cytosol (SOD1), mitochondria (SOD2), and extracellular space (SOD3) [14–16]. After this process, other enzymatic scavengers such as catalases (CATs), peroxiredoxins (PRXs), and glutathione‐disulfide reductases (GSRs) start to cope and neutralize H_2_O_2_. For example, H_2_O_2_ can be converted to H_2_O by oxidizing glutathione [17] to glutathione disulfide (GSSG), while GSR is responsible for converting GSSG to GSH and maintain the glutathione states [18].

Breast cancer is categorized into 5 molecular subtypes according to the expression of 3 receptors (i.e., ER, PR, and HER‐2) and a proliferation factor Ki‐67. These molecular subtypes shall be managed differently with conventional treatments (including surgery, chemotherapy, radiotherapy, hormone‐therapy, and targeted therapy) to reach complete response [19, 20].

In this study, we investigated the anticancer effect of ELF‐EMF on three different breast cancer cell lines with different molecular subtypes, to understand how the mentioned receptors and Ki‐67 affect ELF‐EMF treatment efficiency. Our previous study revealed that the *p53* status has an important role in the glioblastoma cancer cells’ response to ELF‐EMF [21]; hence, together with the ER, PR, HER‐2, and Ki‐67, the *p53* status relation with the apoptosis induction was also investigated. As mentioned above, ROS level and the scavenging system play a pivotal role in cancer cells response to different treatments which target this feature of them. We further assessed the role of ROS level and alteration in the important genes’ expression in the antioxidant machinery after ELF‐EMF exposure.

## 2. Materials and Methods

### 2.1. Cell Lines

Four human breast cell lines were purchased from the Pasteur institute (Iran). Three breast adenocarcinoma cell lines: SKBR‐3 (HER2 enriched), MDA‐MB‐231 (triple‐negative), MCF‐7 (luminal A), and one normal fibroblast cell line were cultured in high glucose‐containing Dulbecco’s modified Eagle medium (DMEM; GIBCO, Thermo Fisher, USA) supplemented with 10% fetal bovine serum (FBS; GIBCO, Thermo Fisher, USA), 100 μg/mL streptomycin, and 100 U/mL penicillin (Invitrogen, Burlington, ON) at 37°C in humidified atmosphere containing 5% CO_2_. Cells (5 × 10^4^) were transferred to 6‐well cell culture plates, and after 24 h of incubation, each of the four cell lines was exposed to 1 Hz, 100 mT ELF‐EMF for 2 h a day for 5 days.

### 2.2. EMF Exposure Systems

The ELF‐EMFs exposure system was comprised of two couples of U‐shape, solenoid‐like configurations with an ironic core coils. Also, the closest distance between the end of the coils is 15 cm. In addition, a magnetic circuit was used to solve the heat problems in the Helmholtz coils systems. The electrically insulated silicon iron sheets, as magnetic core, prohibited any destruction because of eddy current in the core. A Lakeshore G meter was used to point calibration on a net‐like plastic plate located at the bottom of the exposure area. After calibration, a central 8 × 8 cm^2^ was considered as the exposure site where field alteration is less than 2%. The sham exposure group was utilized as the control (all conditions for this group were the same as the exposed ones, except that the sham exposure was not exposed to the ELF‐EMF).

### 2.3. Flow Cytometry Analysis of Apoptosis

In order to evaluate induction of apoptosis following exposure to the ELF‐EMF, all cell lines (fibroblast, SKBR‐3, MDA‐MB‐231, and MCF‐7) were seeded at a density of 5 × 10^4^ cells/well in 6‐well plates and exposed to 1 Hz, 100 mT ELF‐EMF 2 h/day for 5 days. These cells were seeded a day before the exposure was supposed to begin, to let the seeded cells adhere; furthermore, the cells’ density at this step was between 40% and 50%. At the end of the exposure period, cells in early and late stages of apoptosis were stained and detected with FITC‐labeled annexin V/propidium iodide (PI) kit prior to analysis on a Beckman Coulter FC500 instrument (Beckman‐Coulter, Fullerton, CA).

### 2.4. ROS Assessment

The level of ROS following exposure to ELF‐EMF was monitored using the 2′‐7′‐dichlorofluorescin diacetate (DCFH‐DA) assay kit. So, cells were stained with DCFH‐DA green fluorescent dye and then analyzed by a flow cytometry device. Briefly, following ELF‐EMF exposure, culture media were collected and replaced with fresh prewarmed FBS‐free media containing 5 μM DCFH‐DA stains, and incubation continued for 30 min. Subsequently, the supernatant was discarded, and cells were washed three times with chilled PBS. For each experiment, cells were counted and read under the flow cytometer at an excitation wavelength of 488 nm and an emission wavelength of 525 nm.

### 2.5. Quantitative Real Time Polymerase Chain Reaction (qRT‐PCR)

Expression levels of *SOD1*, *SOD2*, *SOD3*, and *GSR* genes following exposure to 1 Hz, 100 mT ELF‐EMF were obtained using the ABI 7500 real‐time PCR system (Applied Biosystems, CA, USA) by SYBR Green PCR Master Mix (Ampliqon, Denmark). In brief, total RNA of exposed and nonexposed cells was extracted with the RNeasy kit (Qiagen, Valencia, CA) based on the manufacturer’s instruction. After this step, complementary DNA (cDNA) was synthesized from the same amount of extracted RNA from different groups using the cDNA Synthesis Kit (AddBio, South Korea). Then, synthesized cDNAs were utilized as templates for the real‐time PCR performed by SYBR Green Master Mix and specific sets of primers (the sequence of primers are provided in Table 1). Real‐time PCR was performed with the following procedure: the first cycle was set at 15 min and 95°C for activation of the enzyme, and 40 other cycles of qRT‐PCR followed: 20 s at 95°C for denaturation of dsDNA and 60 s at 60°C for detecting and reporting fluorescence dye during the annealing and extension step of each cycle. (Human *GAPDH* was used as the internal control for real‐time PCR, and obtained data were analyzed based on the 2^−ΔΔCT^ method).

**TABLE 1:** Sequence of primers used in present study for qRT‐PCR.

Gene name	Sequence (5′–3′)
Superoxide dismutase 1	F: AGTAATGGACCAGTGAAGGTGT
	R: CCAAGTCTCCAACATGCCTCT

Superoxide dismutase 2	F: AGTGTGCGGCACCAGC
	R: CTCGGTGACGTTCAGGTTGT

Superoxide dismutase 3	F: CTCTCTTGGAGGAGCTGGAAAG
	R: CTTGGCGTACATGTCTCGGAT

Glutathione‐disulfide reductase	F: TTCAATGATCAGCACCAACTGC
	R: CCAGAGCAGGCAGTCAACAT

### 2.6. Statistical Analysis

Comparisons between groups were performed with one‐way analysis of variances [22] using GraphPad Prism 8.4.3 software. Throughout the study, data were expressed as the mean ± the standard deviation of the mean (SD). Differences were considered statistically significant with a probability level of *p* < 0.05.

## 3. Results

### 3.1. Flow Cytometry Results

#### 3.1.1. DCFH‐DA Test

**FIGURE 1:**
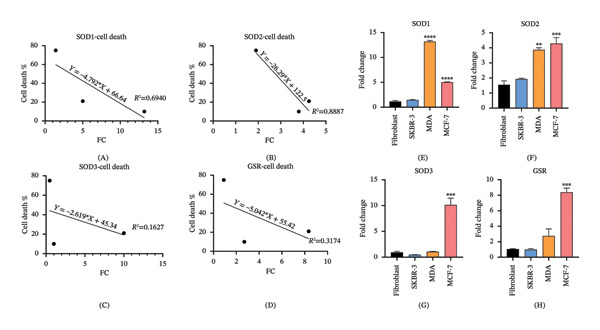
Results of qRT‐PCR after ELF‐EMF treatment (5 days, 2 h/day, 1 Hz, 100 mT) on the expression levels of SODs and GSR genes in studied cell lines. (A) ELF‐EMF exposure on the expression level of SOD1 could not exert significant changes in SKBR‐3 compared to fibroblast, but in MDA‐MB‐231 and MCF‐7 the expression level of this gene significantly increased about 12.5‐fold more (^∗∗∗∗^: *p* < 0.0001) and 5‐fold more (^∗∗∗∗^: *p* < 0.0001), respectively, and the SOD1 expression level in exposed fibroblast cells has no changes compared to the sham‐exposure fibroblast. (B) Exposure of ELF‐EMF on the SKBR‐3 cells showed a nonsignificant increase of about 0.4 fold more in the expression level of the SOD2 gene compared to fibroblasts. The expression level of this gene in MDA‐MB‐231 and MCF‐7 cell lines has been increased meaningfully about 3.9‐fold more (^∗∗^: *p* < 0.01) and 4.2‐fold more (^∗∗∗^: *p* < 0.001), respectively. Also, results show a nonsignificant increase of about 0.5 fold more in exposed fibroblasts compared to its sham‐exposure group. (C) The exposure of ELF‐EMF on SKBR‐3 results in decrease (0.2 fold) in the expression level of SOD3 compared to fibroblast; also, the expression level of this gene did not alter in MDA‐MB‐231 compared to fibroblasts. MCF‐7 showed a significant rise of about 9.9‐fold more in the expression level of SOD3 compared to fibroblast (^∗∗∗^: *p* < 0.001), and fibroblasts have shown no difference between expression levels of SOD3 in exposed and sham‐exposure groups. (D) The effect of ELF‐EMF exposure on the expression level of GSR could not change the expression level of this gene in SKBR‐3 compared to the normal cell line, fibroblast. Also, there was a nonsignificant increase about 3‐fold more in the GSR expression level compared to fibroblast, and GSR expression level has been meaningfully increased about 8.2‐fold more compared to fibroblast in MCF‐7 (^∗∗∗^: *p* < 0.001) (E–H). As we can see, there is a negative correlation between cell death that occurred in cancerous cells and the fold change (FC) of each gene. The best correlation was seen between cell death and the FC of the SOD2 (*R* ^2^ = 0.8887).

**FIGURE 2:**
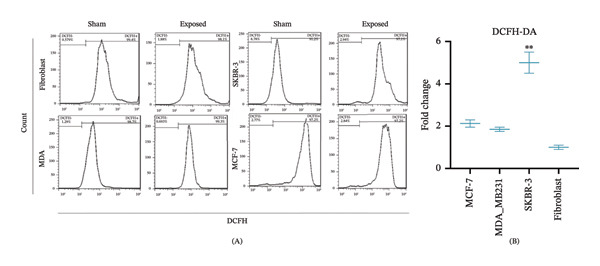
(A) The DCHF‐DA test demonstrated that ELF‐EMF increased the ROS level in exposed groups compared to sham‐exposure groups. (B) Analysis of the DCFH‐DA test shows that ELF‐EMF exposure in SKBR‐3 induced a significant increase of about 5‐fold in the ROS level compared to the fibroblast. On the other hand, induced amounts of ROS in MDA‐MB‐231 (about 1.8 fold) and MCF‐7 (about 1.9 fold) were not statistically meaningful compared to the normal cell line (^∗∗^: *p* < 0.01).

The changes in ROS level were measured by the DCFH‐DA test in exposed and sham‐exposure groups of fibroblast, SKBR‐3, MCF‐7, and MDA‐MB‐231 cell lines. As depicted in Figure 1A, the ROS level increased in all exposed groups compared to their sham‐exposure groups. In Figure 1B, analysis of DCFH‐DA results has demonstrated that the ROS level in SKBR‐3 dropped almost 5‐fold more than in the normal cell line (fibroblast); also, the ROS level increased about 1.8‐ and 1.9‐fold in MDA‐MB‐231 and MCF‐7, respectively, in comparison to fibroblasts. On the other hand, analysis did not show changes between exposed and sham‐exposure groups of fibroblasts in their ROS levels, but importantly, ROS level changes were statistically significant in SKBR‐3 compared to fibroblast (Figure 2).

#### 3.1.2. Annexin V/PI

**FIGURE 3:**
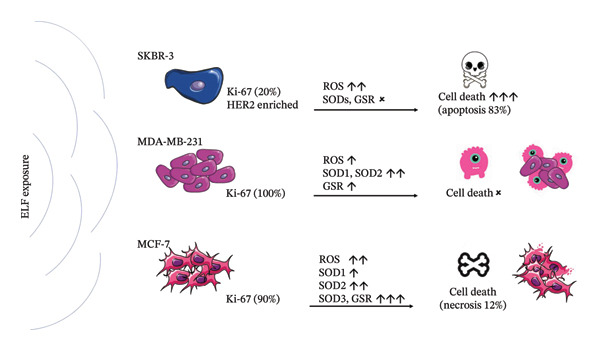
Different breast cancer molecular subtypes respond differently to cell death induction by ELF‐EMF. After ELF‐EMF exposure, ROS level increases in SKBR‐3 cells (HER‐2 enriched, with a low level of Ki‐67), which results in the apoptosis induction in these cells. In MDA‐MB‐231 (triple negative with the high level of Ki‐67) ROS level does not increase as much as the SKBR‐3, after exposure, due to the upregulation of SOD1 and SOD2; the rate of cell death is low in this cell line. In MCF‐7 cells (luminal A, with the high level of Ki‐67 but less than MDA‐MB‐231), ROS level increased after exposure, and the antioxidant machinery (here, mostly GSR and SOD2) could overcome this increase to some levels, which eventually resulted in the apoptosis and necrosis in these cells.

**FIGURE 4:**
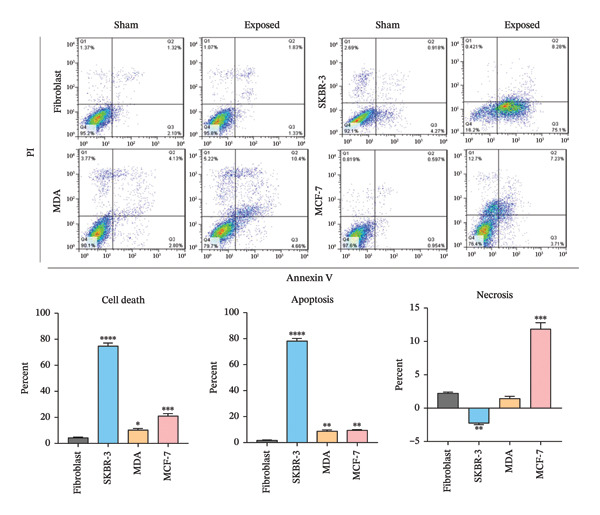
Flow cytometry profile of exposed and sham‐exposed cell lines labeled with annexin V and PI. In fibroblasts, the number of viable cells did not exhibit any obvious alterations after ELF‐EMF treatment (5 days, 2 h/day, 1 Hz, 100 mT) compared to its sham‐exposure groups (3%). Following 5 days of ELF‐EMF treatment in SKBR‐3, the number of annexin V+/PI + cells significantly increased (about 78%; ^∗∗∗∗^: *p* < 0.0001) in comparison to sham‐exposure group (5.6%); but the necrosis percentage dramatically reduced (−2.269%; *p* < 0.001) in the SKBR‐3‐exposed group compared to the nonexposed group. As it is shown, 15% and 6% apoptosis occurred in the MDA‐MB‐231 test and control groups, respectively. Also, 10.9% (^∗∗^: *p* < 0.01) apoptosis occurred in MCF‐7 test group in comparison to control group (1.4%); and a meaningful increase was observed in the percentage of necrosis in MCF‐7, about 12% (^∗∗∗^: *p* < 0.001).

Inhibitory effect of ELF‐EMF exposure (5 days, 2 h/day, 1 Hz, 100 mT) on the cell proliferation of fibroblast, SKBR‐3, MCF‐7, and MDA‐MB‐231 was evaluated by annexin V/PI staining and analyzed by flow cytometry. As shown in Figure 3, this field could effectively reduce the number of live cells in comparison to sham‐exposure groups. Based on the results of flow cytometry, there was no significant difference between the apoptosis of exposed and sham‐exposure groups of fibroblasts with 3% of early and late apoptosis. These results demonstrate a significant increased apoptosis rate in the exposed group of SKBR‐3 (83%) compared to its sham‐exposure group (5.6%). Also, in the MDA‐MBA‐231 cells, the average percentage of apoptosis in exposed and sham‐exposure groups was about 15% and 6%, respectively. The early and late percentage of apoptosis in exposed and sham‐exposure groups of MCF‐7 was about 10.9% and 1.4%, respectively. In addition, MCF‐7 has shown a meaningful increase in the percentage of necrosis, about 12%. Hence, SKBR‐3 represented the highest rate of PCD among the other three studied breast cancer cell lines (Figure 4).

### 3.2. Real‐Time PCR Results

Real‐time PCR was used to investigate the effect of ELF‐EMF (2 h/day, 1 Hz, 100 mT, 5 days) on the expression levels of *SODs* and *GSR* in fibroblasts, SKBR‐3, MDA‐MB‐231, and MCF‐7. As depicted in Figure 1, after exposure, the *SOD1* expression level in SKBR‐3 did not show significant change compared to fibroblast. In MDA‐MB‐231, the *SOD1* expression level increased significantly (about 12.5‐fold) in comparison to fibroblast. Also, the expression level of this gene in MCF‐7 was upregulated significantly (about 5‐fold) compared to fibroblast.

In the case of *SOD2* expression level, ELF‐EMF exposure caused slight increase (about 1.3‐fold) in the expression level of this gene in SKBR‐3, a meaningful upregulation in MDA‐MB‐231 (about 2.5‐fold), and in MCF‐7 (about 2.8‐fold) was seen, compared to the fibroblasts.

The expression level of *SOD3*, in SKBR‐3, and MDA‐MB‐231 did not show significant change in comparison with fibroblast. But the expression level of this mRNA in MCF‐7 it showed a significant upregulation (about 11‐fold) compared to the fibroblasts.

In concurrent with the previous genes, the expression level of the *GSR*, also did not show significant change compared to the fibroblast. In MDA‐MB‐231 the expression level of this mRNA increased about 2.7‐fold, and in MCF‐7 increased about 8.2‐fold compared to the fibroblast.

We further assessed the possible correlation between the expression level of these genes and the cell death occurred in each cell line. All of the four examined genes showed negative correlation with the cell death; among the four examined genes, the SOD2, and then SOD1 revealed the highest correlation (according to their *R* ^2^ score and the negative slope) (Figure 1, and Table 2).

**TABLE 2:** The analysis of flow cytometry and q‐RTPCR results for each cell lines (for more in detail statistical analysis you can see Table S1).

Cell lines	FC ROS^#^	% cell death	SOD1 expression	SOD2 expression	SOD3 expression	GSR expression
Fibroblast	0.8	4	—	—	—	—
SKBR‐3	4.9	75	ns	ns	ns	ns
MDA‐MB231	1.7	10	^∗∗∗∗^	^∗∗^	ns	ns
MCF‐7	1.9	21	^∗∗∗∗^	^∗∗∗^	^∗∗∗^	^∗∗∗^

^#^ROS fold change.

## 4. Discussion

Searching for a new cancer treatment approach which can be utilized alone or as an adjuvant with other current treatment approaches is an undoubted demand according to the ongoing fact that “*cancer is still a socioeconomic dilemma*” [23]. With this regard, various appreciated efforts have been performed to introduce new ways to overcome this chronic disaster. One of the areas which have tried to aid the cancer cure not only with a local and/or holistic point of view but also has tried to increase recipients’ quality of life is renowned as complementary medicine [24–27] (the name that has been delegated from the previous word, alternative medicine, which exchanged into integrative medicine, as the best of both worlds [28]). According to the National Center for Complementary and Integrative Health (NCCIH), EMFs are categorized under physical complementary health approaches. According to the nature of the ELF‐EMF, these fields can affect cancerous cells specifically and hence exert anticancer effects on various tumor types [29, 30]. As they are mostly noninvasive, these fields can be used with other current medicinal approaches with the least, if we do not say so, interaction or interference. One of the main obstacles ahead of these fields that may somehow inhibits them to reach bedside, is that the underlying anticancer mechanism of action of these fields still is not transparent, specifically at cellular and molecular levels. Here, we tried to investigate the relation between PCD induction in breast cancer cells from different molecular subtypes by ELF‐EMF and important receptors (e.g., ER, PR, and HER‐2), proliferation marker (Ki‐67), and *p53* status. We further investigated the role of ROS level (as an emerging proposed mechanism of action of these fields [4]) and ROS scavenging system in the response of these cancer cells to the ELF‐EMF.

### 4.1. Relation Between Breast Cancer Molecular Subtypes and ELF‐EMF PCD Induction

To study this relation, three breast cancer molecular subtypes including MCF‐7 (luminal A), SKBR‐3 (HER‐2 enriched), and MDA‐MB‐231 (triple negative) were exposed to the ELF‐EMF. According to the surface receptors, HER‐2 plays a pivotal role in the PCD induction after ELF‐EMF exposure; the rate of apoptosis induction in the SKBR‐3 is about 83%, which is much more compared with the other cell lines. The next step is MCF‐7 (ER and PR positive); however, in this cell line, apoptosis and necrosis occurred after exposure. This result is aligned with our previous study, in that we demonstrated that ELF‐EMF (1 Hz, 100 mT) could increase phosphorylation of RIPK1/RIPK3/MLKL proteins and cleavage of caspase‐9/caspase‐3, which finally resulted in necroptosis and apoptosis, respectively, in the MC4L2 cells [31] (the cell surface receptors of MC4L2 and MCF‐7 are so similar). The least PCD induction occurred in the triple negative subtype (MDA‐MB‐231). These results are in accordance with previous research by Loredana Bergandi et al. that showed ELF‐EMF exposure (8 Hz, 4 days) causes more cell death in treated SKBR3 compared to the treated MCF‐7 [32]. Furthermore, other research about the effect of ELF‐EMF (50 Hz, 12 μT/7 days) on the different types of cancer cell lines (SKBR‐3, GTL16, HT29, and A375P) indicated that ELF‐EMF exposure causes more cell death in the treated SKBR‐3 cells [33].

Since the overproduction of HER2 causes high activation of the mechanistic target of mTOR [34], importantly, it is not completely understood whether ELF‐EMF just blocks the HER‐2 proteins dimerization or affects the downstream cascade AKT/mTORC. In cancer cells, one of the mainstream impacts that are proposed for ELF‐EMFs cell death induction is believed to be through ROS elevation [35–37]. Furthermore, accumulation of ROS can inhibit mTOR and PI3K/AKT pathways which lead to the autophagy [38]. Also, ELF‐EMF (120 Hz, 4.5 mT) significantly decreased cyclin D1 expression level (another factor that was affected by HER2 activation) in the rat hepatocarcinogenesis [39].

From the Ki‐67 point of view, this factor plays as an indicator in breast cancer classification, prognosis, and prediction of therapeutic responses; furthermore, it represents the proliferative activity of breast cancer cells [40]. This proliferation biomarker has a negative correlation with the ELF‐EMF apoptosis induction; the apoptosis induction is the most in the SKBR‐3 with the least Ki‐67 expression level [39]. Also, ELF‐EMF (120 Hz, 4.5 mT) leads to a reduction in the level of Ki‐67 and results in decreased cell proliferation of preneoplastic lesion in hepatocarcinogenesis in male Fischer‐344 rat model [39]. As mentioned above, one of the main cell death‐related pathways that may occur after ELF‐EMF exposure is dependent on the apoptosis induction through caspase‐3 activation. It is reported that downregulation of Ki‐67 can induce caspase‐3 activation and subsequently apoptosis in breast cancer cells [41, 42], hence it is not out of mind to expect that the apoptosis induction shall be higher in the breast cancer cells with lower Ki‐67 levels.

*p53* status (i.e., wild‐type or mutant) may respond differently to therapies that are dependent on the downstream *p53* death signaling pathways [1, 43]. It is reported that apoptosis induction by electrical fields (siblings for the magnetic fields) is due to the *p53* status [44, 45], hence, here we investigated the possible relation between this gene status and the cell death and/or apoptosis rate in the three breast cancer cell lines after being exposed to ELF‐EMF. SKBR‐3 and MDA‐MB‐231 cells harbor a mutation in the p53 gene but MCF‐7 has the wild‐type of this gene [46]. In contrast with our previous study and previously reported results, where we revealed that the *p53* status plays a crucial role in the apoptosis induction of ELF‐EMF in glioblastoma cancer cells [21, 43], here, it seems that in breast cancer, the *p53* status is not so critical; as we can see the most apoptosis induction occurred in the cell line which suffers from *p53* mutant (Table 3). It may be considered that the cell‐death induction by ELF‐EMF is not dependent on *p53* status in breast cancer cells. This result may be somehow explained by Destefanis et al., who demonstrated that ELF‐EMF (50 Hz, 12 μT) could not alter the total expression and nuclear level of *p53*, while the mitochondrial level of it decreased in SKBR‐3 as the consequence of increased permeability of mitochondria that can occur post exposure [33].

**TABLE 3:** The characteristics of studied breast cancer cell lines.

Cell line	ER	PR	HER‐2	% ki‐67	p53 status	% apoptosis	% necrosis
SKBR‐3 (HER‐2)	0	0	3+	20	Mutant	78	0
MDA‐MB231 (Basal)	0	0	0‐1+	100	Mutant	9	2
MCF‐7 (luminal A)	6	6	0‐1+	90	WT	10	12

### 4.2. Relation Between ROS Level/Scavenging System and ELF‐EMF PCD Induction

One of the mainstream mechanisms proposed for ELF‐EMFs cell death induction in cancer cells is believed to be through ROS elevation [35–37], especially superoxide anion levels [47]. It has to be noted that Nrf2 is an important antioxidant response element (ARE), and its expression is affected after ROS elevation therapies like magnetic fields [48]. Subsequently, Nrf2 regulates the expression of antioxidant enzymes in response to increased ROS levels or oxidative stress conditions [49, 50]. Furthermore, not only is ROS elevation a crucial criterion in cell‐death induction by ELF‐EMFs, but also the genetic pattern of cells plays a pivotal role in this area. ROS scavenger genes such as SODs and GSR are touchstones that determine cell fate in this regard [6, 14]. SODs as the first line of antioxidant enzymes compete with nitric oxide (NO) and reduce superoxide anions to H_2_O_2_ then allow other scavengers to catalyze it to H_2_O [15]. Our results revealed that although ROS accumulation and more apoptosis induction have occurred in SKBR‐3 after ELF‐EMF exposure, SODs and GSR expression levels did not show a significant change in this cell line. According to the therapeutic window of ROS levels, cancerous cells are expected to be more susceptible to the ROS elevation and undergo apoptosis than normal cells [51]; furthermore, SKBR‐3 did not show resistance to the ROS elevation by endogenously upregulation of the mentioned scavengers which resulted in a high apoptosis rate in this cell line.

On the other hand, in MDA‐MB‐231, the expression of SOD1 and SOD2 increased after the exposure which can neutralize superoxide anion in favor of maintaining redox balance and therapy resistance to the treatment seen in this cell line. Among the three cancerous cell lines, MDA‐MB‐231 showed the most resistance against cellular death induced by ELF‐EMF; from the ROS scavenger genes point of view, we see that the expression of the SOD1 gene was much higher in this cell line compared with the others. This may reflect the importance of SOD1 in the resistance of this cell line against ELF‐EMF. This phenomenon can be explained even partially with the role and localization of the product of this gene: the SOD1 enzyme localizes at the cytoplasm [14], where it is reported to be the main area for the ROS‐elevating occurrence after ELF‐EMF exposure [6, 52]. Therefore, our results suggest that ELF‐EMF–dependent cell death can occur in MDA‐MB‐231 with effective gene silencing of SOD1 because almost all O_2_ production sources release it into the cytoplasm, where SOD1 is localized.

## 5. Conclusion

Different breast cancer molecular subtypes respond uniquely when they are exposed to ELF‐EMFs, but ROS elevation is the main reason which shall guide cells towards death (the HER‐2‐enriched subtype, with lower Ki‐67, seems to be more susceptible to undergoing death). In this area, the antioxidant machinery plays a pivotal role; if this machine (especially SOD1) could adapt itself to the ROS elevation, the rate of cellular death after exposure would be the least. This scenario proposed that ELF‐EMF can be recruited in adjuvant modalities which target HER‐2 and/or induce ROS elevation.

## Author Contributions

Mohadeseh Shayeghan, Fatemeh Afroughi, and Fatemeh Shahriari performed the laboratory experiments and first draft of the manuscript; Flora Forouzesh and Mohammad Hadi Niakan contributed in analyzing the obtained data, Mohammad Amin Javidi conceived the main idea of this study; Mohammad Amin Javidi supervised the study.

## Funding

No funding was received for this manuscript.

## Disclosure

All the authors reviewed the manuscript.

## Consent

All of the authors give their consent for publication of the identifiable details of the text.

## Conflicts of Interest

The authors declare no conflicts of interest.

## Supporting Information

Additional supporting information can be found online in the Supporting Information section.

## Supporting information


**Supporting Information** In detail statistical analysis.

## Data Availability

The data that support the findings of this study are available from the corresponding authors upon reasonable request.
