# Supplementary material for: Pivotal Factors in Breast Cancer Molecular Subtypes Apoptosis Induction by ELF‐EMF; Ki‐67, ROS Level, HER‐2, and SODs
Source: Breast J. 2026 Jul 17;2026:9572421. doi: 10.1155/tbj/9572421 (PMC13378569; doi:10.1155/tbj/9572421)
Supplement: Supplementary file 1 — Supporting Information In detail statistical analysis. [file TBJ-2026-9572421-s001.pptx]

## Slide 1
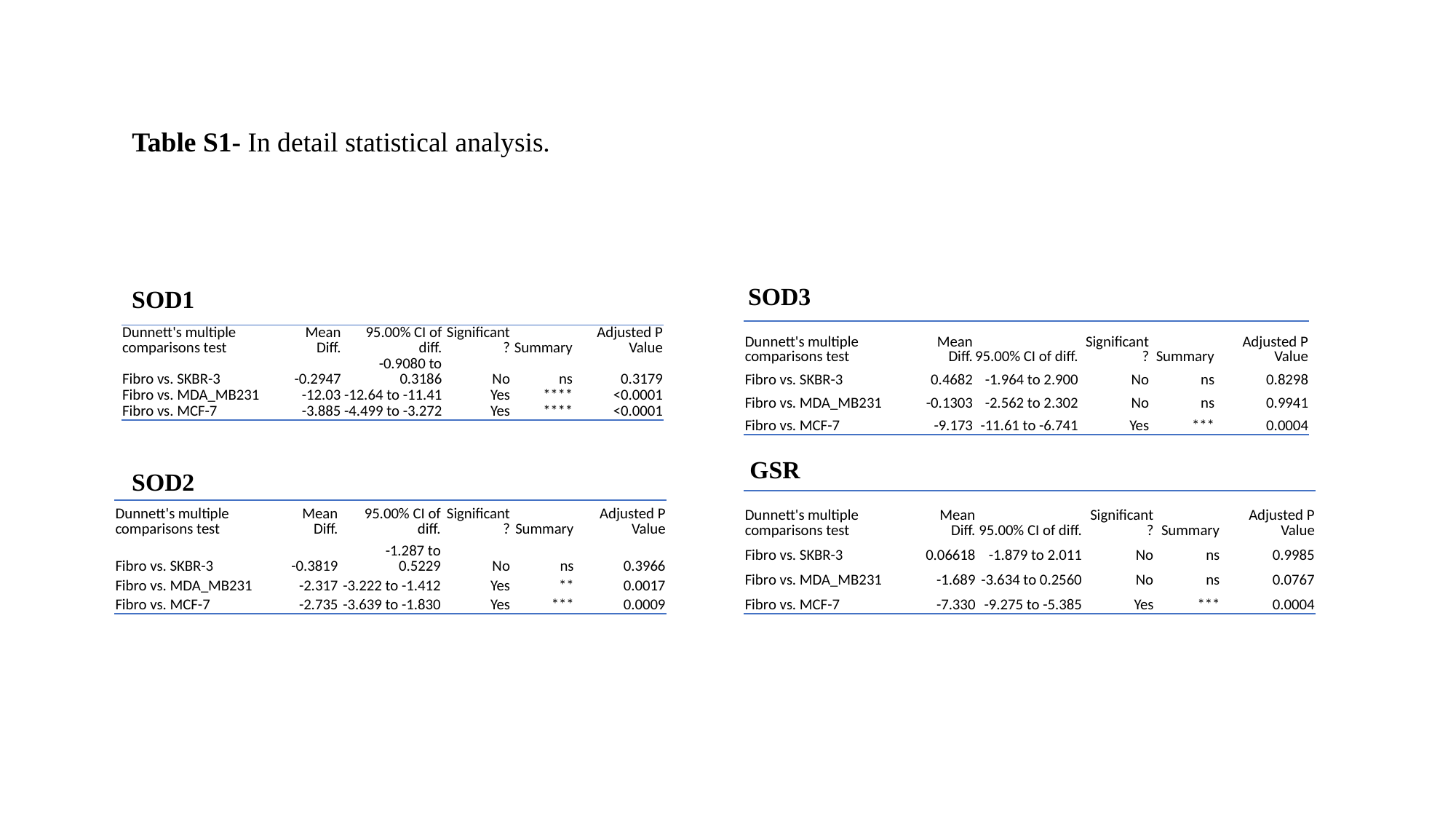

Table S1- In detail statistical analysis.
SOD3
SOD1
| Dunnett's multiple comparisons test | Mean Diff. | 95.00% CI of diff. | Significant? | Summary | Adjusted P Value |
| --- | --- | --- | --- | --- | --- |
| Fibro vs. SKBR-3 | 0.4682 | -1.964 to 2.900 | No | ns | 0.8298 |
| Fibro vs. MDA\_MB231 | -0.1303 | -2.562 to 2.302 | No | ns | 0.9941 |
| Fibro vs. MCF-7 | -9.173 | -11.61 to -6.741 | Yes | \*\*\* | 0.0004 |
| Dunnett's multiple comparisons test | Mean Diff. | 95.00% CI of diff. | Significant? | Summary | Adjusted P Value |
| --- | --- | --- | --- | --- | --- |
| Fibro vs. SKBR-3 | -0.2947 | -0.9080 to 0.3186 | No | ns | 0.3179 |
| Fibro vs. MDA\_MB231 | -12.03 | -12.64 to -11.41 | Yes | \*\*\*\* | <0.0001 |
| Fibro vs. MCF-7 | -3.885 | -4.499 to -3.272 | Yes | \*\*\*\* | <0.0001 |
GSR
SOD2
| Dunnett's multiple comparisons test | Mean Diff. | 95.00% CI of diff. | Significant? | Summary | Adjusted P Value |
| --- | --- | --- | --- | --- | --- |
| Fibro vs. SKBR-3 | 0.06618 | -1.879 to 2.011 | No | ns | 0.9985 |
| Fibro vs. MDA\_MB231 | -1.689 | -3.634 to 0.2560 | No | ns | 0.0767 |
| Fibro vs. MCF-7 | -7.330 | -9.275 to -5.385 | Yes | \*\*\* | 0.0004 |
| Dunnett's multiple comparisons test | Mean Diff. | 95.00% CI of diff. | Significant? | Summary | Adjusted P Value |
| --- | --- | --- | --- | --- | --- |
| Fibro vs. SKBR-3 | -0.3819 | -1.287 to 0.5229 | No | ns | 0.3966 |
| Fibro vs. MDA\_MB231 | -2.317 | -3.222 to -1.412 | Yes | \*\* | 0.0017 |
| Fibro vs. MCF-7 | -2.735 | -3.639 to -1.830 | Yes | \*\*\* | 0.0009 |
